# Depressive Anxiety Symptoms in Hospitalized Children with Chronic Illness during the First Italian COVID-19 Lockdown

**DOI:** 10.3390/children9081156

**Published:** 2022-08-02

**Authors:** Cinzia Correale, Chiara Falamesca, Ilaria Tondo, Marta Borgi, Francesca Cirulli, Mauro Truglio, Oriana Papa, Laura Vagnoli, Cinzia Arzilli, Cristina Venturino, Michele Pellegrini, Valentina Manfredi, Rossella Sterpone, Teresa Grimaldi Capitello, Simonetta Gentile, Simona Cappelletti

**Affiliations:** 1 Clinical Psychology Unit, IRCCS Bambino Gesù Children Hospital, 00146 Rome, Italy; chiara.falamesca@opbg.net (C.F.); ilaria.tondo@opbg.net (I.T.); teresa.grimaldi@opbg.net (T.G.C.); simona.cappelletti@opbg.net (S.C.); 2 Center for Behavioral Sciences and Mental Health, Istituto Superiore di Sanità, 00161 Rome, Italy; marta.borgi@iss.it (M.B.); francesca.cirulli@iss.it (F.C.); 3 School of Fundamental Sciences, Massey University, Palmerston North 4442, New Zealand; mauro.truglio@gmail.com; 4 Children’s Neuropsychiatric Ward, Regional Pediatric Hospital “G. Salesi”, 60123 Ancona, Italy; oriana.papa@ospedaliriuniti.marche.it; 5 Pediatric Psychology, Meyer Children’s Hospital, 50139 Florence, Italy; laura.vagnoli@meyer.it (L.V.); cinzia.arzilli@meyer.it (C.A.); 6 Psychology Unit, IRCCS Istituto Giannina Gaslini, 16147 Genoa, Italy; cristinaventurino@gaslini.org; 7 Azienda Ospedaliera Universitaria Policlinico—Giovanni XXIII, 70124 Bari, Italy; michele.pellegrini@policlinico.ba.it; 8 Psychology Unit, A.O. S.S. Antonio Biagio and C. Arrigo Hospital, 15121 Alessandria, Italy; valentina.manfredi@ospedale.al.it (V.M.); rsterpone@ospedale.al.it (R.S.); 9 Department of Humanities, LUMSA University, 00193 Rome, Italy; s.gentile6@lumsa.it

**Keywords:** COVID-19, lockdown, mood disorders, chronic illness, anxiety, depression, pandemic, mental health, childhood

## Abstract

COVID-19 is continuing to spread around the world, having a direct impact on people’s daily lives and health. Although the knowledge of the impact of the COVID-19 pandemic on mental health in the general population is now well established, there is less information on its effect on specific and vulnerable populations, such as children with chronic illness (CI). We conducted a multi-centered cross-sectional study among pediatric patients in six public children’s hospitals in Italy during the first lockdown, with the aim of assessing the proportion of children with CI presenting anxiety and depressive symptoms, and the clinical and demographic characteristics affecting such symptomatology. We included children with at least one chronic condition, with no cognitive delay, aged between 11 and 18 years. Brief standardized questionnaires were administered during medical scheduled visits to screen anxiety and depressive symptoms. We found a very high proportion of children showing mild to severe depressive and anxiety symptomatology (approximately 68% and 63%, respectively). Our results highlight the need of ensuring tailored psychological interventions to protect children with CI from the effect of the pandemic (and related restrictive measures such as quarantine and social distancing), with the final aim of promoting mental health and psychological well-being in this vulnerable population.

## 1. Introduction

Although more than two years have passed since the first case, the COVID-19 pandemic is still ongoing across most of the world’s populations, having a direct impact on people’s lives and health [1]. Although the knowledge of the effects of the COVID-19 pandemic on mental health in the general population is now well established, less is known about its impact on specific and vulnerable populations. This information is particularly important to further enhance the potential to prepare for future epidemics and pandemics, as well as providing targeted intervention and support measures for particularly affected groups, such as children with chronic illness [2].

Chronic illness (CI) in childhood is a heterogeneous category encompassing diverse diseases with varying degrees of impact on children and their families [3,4]. Prevalence rates of CI in children vary greatly, ranging from 3.5% to 35.3% [5]. Rates of childhood CI have steadily increased from 1.8% in 1960 to 7% in 2004, mostly as a result of advances in healthcare, which have allowed children to live longer [6], as well as of changes in diagnostic processes and management. The diagnosis of CI is often associated with multimorbidity (e.g., IBD and psoriatic arthritis) and can have a life-long impact on children’s well-being and quality of life, leading to a greater risk of developing psychological and mental problems, especially mood disorders [7,8,9].

As a result of the COVID-19 pandemic, a substantial increase in the prevalence and burden of major depressive and anxiety disorders has been observed in the general population [10,11,12]. Social restrictions, lockdowns, school and business closures, loss of income, and the shifting priorities of governments in their attempts to control COVID-19 outbreaks all have negatively affected people’s mental health. Emerging evidence on the impact of the coronavirus (COVID-19) pandemic shows that young people are among the groups who are disproportionately affected by the current pandemic, particularly in terms of mental well-being, education, training, and employment. As a result, higher rates of anxiety and depression have been observed, as shown by a meta-analysis on the global prevalence of depressive and anxiety symptoms in children and adolescents during COVID-19 [13]. The spread of the disease and social distancing measures have even more strongly impacted vulnerable target groups with pre-existing mental health conditions or those already facing social integration barriers and daily routine disruption, such as children with CI. 

Moreover, during the COVID-19 pandemic, this population had to face the interruption—or delay—of medical assistance, hospital treatment, and diagnostic procedures. In Italy, one of the countries most affected by COVID-19, Lazzerini and colleagues [14] reported 12 pediatric cases of delayed access to hospital care during the week of 23–27 March 2020 across five hospitals. Of these cases, half were admitted to an Intensive Care Unit and four died of medical diseases that, if treated in time, could have had a better outcome. All patients were affected by a CI and parents reported delaying access to care because of concerns about being exposed to COVID-19 infection (fear of contagion). Considering that a family with a child with CI commonly suffers a significant burden of care associated with financial and social problems, the COVID-19 pandemic has exacerbated the gap between the family’s needs and the healthcare provided. As an example, Zhang and colleagues [15] conducted research among a group of parents of children with Chronic Kidney Disease (CKD) in China, confirming that 62.3% of parents were worried that the CKD might relapse or be aggravated during the pandemic since they could not regularly see a doctor.

Notwithstanding the very first recommendations promoted by several scientific and academic committees to address particular attention to vulnerable groups [16], to date, we have still little information on how the COVID-pandemic has affected the mental health of children with CI. 

We conducted a multi-centered cross-sectional study among pediatric patients in six public children’s hospitals in Italy, with the aim of exploring the manifestations of anxiety and depression symptomatology among children with CI in the very first phase of the Italian lockdown. 

## 2. Materials and Methods

We conducted a multi-centered cross-sectional study among pediatric patients in six public children’s hospitals in Italy (Table 1). We collected data from 3 March 2020 to 17 May 2021, performing face-to-face interviews during scheduled medical examinations (day hospital, hospitalization, and follow-up), medical follow-up teleconsultation, or via online survey forms. Upon completion of the evaluation interviews, the questionnaires were delivered and scored by a qualified clinical psychologist of the Clinical Psychology Unit (CPU). The clinical psychologist then recorded the results in the appropriate CRF and entered the values into the database, following the appropriate de-identification strategy. The response rate for this study was roughly 100%, since the delivery of the questionnaires took place before or during the interview with the clinical psychologist, who made sure, before the resignation, that the patient had completed the whole questionnaire. This was also true in the few instances where questionnaires were delivered through Google modules. If the questionnaire was not delivered by the patient, it was completed by the clinical psychologist via a telephone interview.

### 2.1. Participants

Starting from the more conservative hypothesis of a prevalence of 50%, we calculated that a sample of at least 380 individuals would allow us to estimate the prevalence of psychological discomfort with a margin of error of 10% and a confidence level of 5% (95% confidence interval). Then, we were able to recruit a total sample of 334 children (60% F) aged between 11 and 18 years (mean 14.44; SD 1.85). The inclusion criterion was children with at least one chronic condition, while the exclusion criterion was children with cognitive delay (IQ ≤ 85). Participants were recruited through the Clinical Psychology Units of the children’s hospitals participating in the project, and the CPU’s personnel carried out assessments during routine or scheduled examinations. Each participant spent no more than 10 min approximately in completing both questionnaires. Table 2 describes the clinical characteristics of the study sample. 

### 2.2. Measures

In order to assess anxious–depressive symptoms, we administered the Generalized Anxiety Disorder scale (GAD-7) [17] and the Patient Health Questionnaire (PHQ-9) [18,19,20,21]. The Generalized Anxiety Disorder (GAD-7) scale measures seven symptoms. Participants are asked how often they have experienced each symptom in the past two weeks. The response options are “not at all,” “several days,” “more than half the days,” and “nearly every day,” scored as 0, 1, 2, and 3, respectively. The thresholds for symptom severity are: 5–9 mild, 10–14 moderate, and 15–21 severe. This tool has been used in a variety of studies to assess anxiety symptoms in a wide range of chronic conditions [22,23]. The PHQ-9 is a simple, very effective self-assessment test for depressive symptomatology. Participants are asked to indicate the presence of nine issues, including depression and interest decline, in the last 2 weeks on a 4-point scale ranging from “nearly every day” (3 points) to “not at all” (0 points). The thresholds for symptom severity are: 5–9 mild, 10–14 moderate, 15–19 moderately severe, and 20–27 severe. The PHQ-9 has good internal consistency, with a Cronbach’s alpha coefficient between 0.80 and 0.90. Both the PHQ-9 and GAD-7 measures are suitable for use with persons 11 years of age and older.

### 2.3. Data Analysis

All the statistical analyses were performed using JASP, R [24,25], and custom Python scripts. The analysis was organized into two stages. For the first one, our sample was divided into subgroups according to various parameters (clinical, i.e., disease-specific groups, and demographic) and Wilcoxon’s signed-rank one-sample test was used to assess whether PHQ-9 and GAD-7 scores were substantially different from the threshold score of 5 for any symptomatology. For the second stage, we carried out comparisons between said subgroups, using Mann–Whitney’s U test and Kruskal–Wallis with Dunn’s post-hoc test with Bonferroni–Holm correction. The only exception was the “continuity of care during COVID-19′s first lockdown” variable, where we used Welch’s *t*-test as the dataset was unbalanced. All the correlations were calculated using Spearman’s Rho. The significance threshold for all tests was set at *p* < 0.05.

## 3. Results

Table 3 shows the proportions of patients with different levels of depressive and anxiety symptoms. Mild and moderate depressive and anxiety symptoms were most common. The rate of mild depression was 36.23%, while that of moderate depression was 23.65%; meanwhile, the rate of mild anxiety was 33.53%, and that of moderate anxiety was 19.76%. The proportion of all of the participating patients with mild-to-severe depressive symptoms was 67.96%, and the proportion of all of the patients with mild-to-severe anxiety was 62.87%. The prevalence of comorbid depressive and anxiety symptoms was 53.89% among all the participants.

Table 4 shows that, according to the Wilcoxon one-sample test, patients diagnosed with migraines, diabetes, and gastrointestinal diseases had statistically significant rates of anxious symptomatology compared to the mild-to-severe threshold of 5, while only migraines and diabetes showed statistically significantly higher rates of depressive symptomatology than the mild-to-severe threshold of 5. 

The overall sample of children with CI showed strong statistically significant anxiety and depressive scores and symptomatology (*p*-value < 0.001) (Table 5). Females exhibited a significantly higher mean score for anxious and depressive symptoms (*p*-value < 0.001) while males did not. Both patients receiving and not receiving pharmacological treatment showed statistically significant scores for depressive and anxiety symptomatology. Similarly, all patients following and not following non-pharmacological therapies (i.e., psychotherapy, speech therapy, etc.) showed very high scores for mood symptoms (*p*-value < 0.001). Among patients receiving continuity of care during the COVID-19 lockdown and suspension of deferrable medical examinations, both groups showed significantly elevated rates of mood disorders, even if those who had had the opportunity to see a doctor showed less statistically significant scores of mood impairment (*p*-value < 0.01) than those who suspended visits (*p*-value < 0.001). In terms of years of illness, again, the overall group showed statistically significant scores of mood impairment, even if the group of patients with ≥5 years of illness showed the lower statistical significance score for depression (*p*-value < 0.05). Finally, children diagnosed with CI at school had statistically significant depression and anxiety symptomatology scores, whereas those diagnosed in preschool showed average rates below the clinical cut-off.

Next, we found that, as years of illness increase, there is a downward trend in anxiety and depression scores, even if it is not statistically significant (anticorrelation −0.140). Moreover, depressive–anxious scores do not depend on the patient’s age.

Next, we were interested in investigating whether some variables had an impact on depressive–anxious symptoms. 

**Figure 1:**
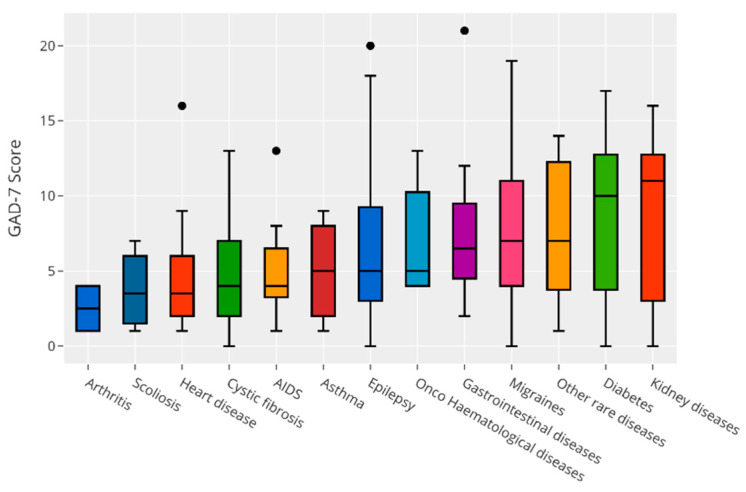
Relationship between anxiety scores and single diagnosis of CI.

**Figure 2:**
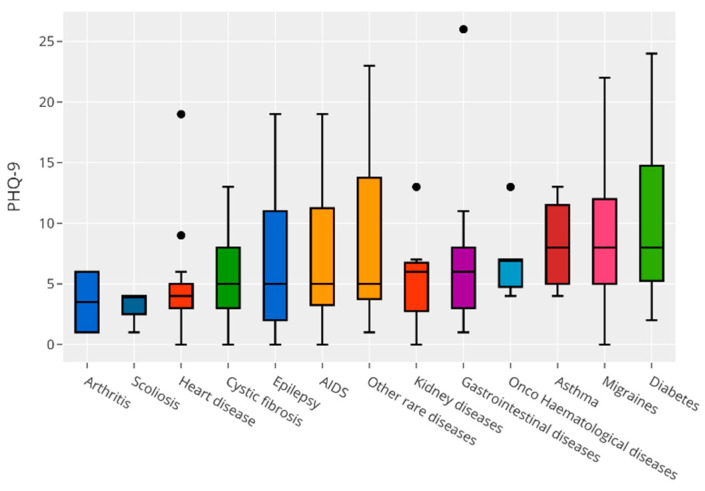
Relationship between depression scores and single diagnosis of CI.

Figure 1 and Figure 2 present the relationship between anxiety and depression scores and single diagnosis of CI. The Kruskal–Wallis test found no significant difference in scores between diagnoses, so we could conclude that there was no precise diagnosis that influenced mood scores (data omitted).

Then, we proceeded to investigate whether there was a statistically significant difference in mood scores between sub-groups (Table 6), using Mann–Whitney’s and Kruskal–Wallis’ tests. We were interested in investigating the effect of children’s demographic and clinical characteristics, such as age at diagnosis (preschool vs. school age), being under pharmacological therapy (yes or not), being under non-pharmacological treatment (speech therapy, psychotherapy, etc., or not), having continuity of care while in lockdown or restriction period, and sex, on depressive and anxiety symptoms. No differences between groups were found, except for age at diagnosis and sex. More specifically, children who received the diagnosis of CI during school age and girls had statistically significantly higher scores of depressive symptoms than children who were diagnosed during preschool and boys. 

Lastly, we found that anxiety and depression scores were strongly correlated (rh0 = 0.671, *p* < 0.001)

## 4. Discussion

The aim of the present study was to assess the proportion of children with CI presenting anxiety and depressive symptoms, and the clinical and demographic characteristics affecting such symptomatology.

Depressive and anxiety symptomatology was assessed using standardized questionnaires largely used in populations with chronic conditions [26]. Our study population represents various regions in Italy, although the majority resided in the central area of the country and were affected by a wide variety of chronic conditions. The majority of the participants were adolescents or pre-adolescents (mean age 14.44, range 11–18) and female (201F; 133M).

We found a very high proportion of children showing mild to severe depressive and anxiety symptomatology (approximately 68% and 63%, respectively). No differences were found among children affected by different CI, although the lack of significance may be due to the small sample size. Future studies with larger sample sizes should investigate whether children with specific conditions are more at risk of mental health issues.

Concerning the pharmacological and other (non-pharmacological) therapies, again, very high scores of anxiety and depression symptomatology were found in those receiving or not receiving the treatment, but no statistical differences were found between groups. Regarding the impact of the duration of the chronic condition (years of illness), our analysis showed an inverse downward trend—the longer the years of the disease, the lower the levels of anxiety and depression—even if this result did not reach statistical significance. This finding is consistent with previous literature, presuming a process of long-term adaptation to the disease [27].

Regarding the sex, girls in our sample showed significantly higher scores of symptoms, both anxiety and depression, than boys. This has been already documented in the general population [28], but is in contrast with previous findings in some chronic conditions [29,30,31]. Further research should better understand the factors involved in differentiating the symptomatology between males and females, related also to the agreement between self-report measures and proxy reports.

Lastly, we found high levels of mood disorders only in children who had been diagnosed with CI at school age, a result confirmed by a statistically significant correlation between the age at diagnosis and depression scores. These results could be due to the processes of adaptation to the illness, which can be enhanced also by early detection and diagnosis of the disease, where possible. In addition, timely detection and communication of diagnosis can be used to promote early coping strategies in patients with chronic illness, and to buffer some disease-related outcomes, such as behavioral problems [32].

### 4.1. Depression and Anxiety Manifestations and Proportions

Mental disorders are increasingly identified as the main cause of the burden of illness in the general population [33]. According to the Global Burden of Diseases, Injuries, and Risk Factors Study 2019 (GBD), between 1990 and 2019, mental health disorders increased by 48.1% worldwide, with the two most common conditions represented by depressive and anxiety disorders [1]. A recent review conducted by LaGrant and colleagues (2020) found that, among children with epilepsy and other chronic conditions living in the United States during the pre-pandemic period, 25% of children aged 5–17 with epilepsy and 22.1% of children with other conditions had depression and/or anxiety symptoms [31].

The authors of this study note that many previous studies have estimated the prevalence of depression and anxiety in children suffering from chronic illness [34,35,36]: in the case of epilepsy, estimates vary widely between studies—rates of depression range from 8 to 33%, while rates of anxiety range from 5 to 48.5%. Regarding comorbidity, 11.5% of all children with epilepsy had both depression and anxiety. For other chronic conditions, such as asthma, migraines, and allergies, reported overall rates of depression range from 2.8% to 7%, while rates of anxiety range from 7.5% to 15.8%. Comorbidity among these conditions ranges from 6.2% to 20.4%. When compared to these data, we found far greater proportions among our sample: depressive symptoms at 67.96% and anxiety symptoms at 62.87%, with a prevalence of comorbidity of 53.89%.

According to these results and considerations, the COVID-19 pandemic appears to have exacerbated the psychological burden on this population, but it is not limited to this group. Indeed, a recent systematic review conducted by Ma and colleagues (2021) [37] found a pooled prevalence of depression among children of 29% (ranging from 10% to 71%) and a pooled prevalence of anxiety of 26% (ranging from 7% to 55%) during the COVID-19 pandemic.

However, further investigations are needed to obtain a clearer picture of children with chronic illness worldwide. It is important to note that during the COVID-19 pandemic, school closures were one of the first containment measures implemented by governments around the world. School is an important social and support resource for children with chronic conditions, which provides rehabilitation interventions, special learning programs, coping routines, and socialization among peers. As stated by Lee [13], social restrictions exposed high-risk populations, such as pediatric CI patients, to the onset or relapse of anxious–depressive symptoms.

Even though our study has some limitations, such as a more representative sample from Central Italy and fewer from other regions, these preliminary data show alarming percentages of depressive and anxiety symptoms, calling for an urgent effort to implement tailored prevention and public treatment programs addressing the needs of this vulnerable group of patients.

### 4.2. Continuity of Care

Of the overall sample of 334 children, only 35 of them (10,48%) received continuity of care, while 293 children (89,52%) did not. In both groups, we found very high levels of depressive and anxiety symptoms, which are consistent with previous findings showing children with CI as an “at-risk” group for mood impairment [38]. Even if no statistical significance was reached, lower levels of mood symptomatology were found in the group of patients receiving continuity of care during the early phase of the COVID-19 pandemic in Italy, suggesting the role that continuity of care may play as a protective factor for the emergence of mental disorders.

The COVID-19 pandemic caused the disruption of medical essential services and delayed care-seeking due to fear of contracting the virus [14]. Guaranteeing continuity of care is a critical factor, not only to provide stable monitoring and managing of their chronic condition, to prevent relapses, and to ensure medical prescriptions, but also to promote mental health and ensure resilience in this population. Resilience implies having skills that can help a person facing (and coping with) stressful/threatening events. The presence of risk and protective factors have a strong impact in the promotion of positive outcomes, as well as in the reduction of the negative ones [39].

The “state of emergency” caused by the COVID-19 pandemic focused on hospital care to prevent the health system from being over-burdened, overlooking the importance of primary care in guaranteeing continuity of care. This contributed to a rupture and loss of references and services, essential to promote the sense of safety and protection that the chronic patient and his/her family can benefit from. Ensuring continuity of care during critical times, especially for those in vulnerable categories such as children with CI, should be prioritized also as an inherent path to promote resilience and mental health. As Prof. Prince and colleagues (2007) stated in their famous article published in the prestigious journal “The Lancet”, “There is no health without mental health” [40].

## 5. Conclusions

Our analysis showed a very high proportion of anxious–depressive symptomatology in a sample of children and adolescents with CI during the first COVID-19 lockdown in Italy. As the pandemic is still ongoing, continuity of care must be ensured and tailored psychological assistance should be enhanced in order to promote and manage their mental health. Further longitudinal and larger studies are still needed to obtain deeper knowledge of the phenomenon and related risk/protective factors.

## Figures and Tables

**Table 1:** Participants’ sample size: patients per center (*n* = 334).

Pediatric Center	Patients (*n*)
IRCCS O.P. Bambino Gesù	242
IRCCS Istituto Giannina Gaslini	5
O.P. Giovanni XXIII	17
A.O.U. Meyer	35
A.O.U. Ospedali Riuniti di Ancona	28
A.O. SS. Antonio e Biagio e C. Arrigo	7

IRCCS: Istituto di Ricovero e Cura a Carattere Scientifico; O.P.: Ospedale Pediatrico; A.O.U.: Azienza Ospedaliera Universitaria; A.O.: Azienda Ospedaliera.

**Table 2:** Clinical characteristics (CI diagnosis) of the study sample (*n* = 334).

CI Diagnosis		*n*
Epilepsy		53
	Generalized	10
	Focal	34
	Others	9
Heart Disease		14
	Fontan	6
	Cardiomyopathy Hypertrophic	1
	Interventricular Defect	3
	Defect Transposition of Large Vessels	1
	Congenital Cardiopathy	2
	Third-Degree Ventricular Atrium Block	1
AIDS		16
Cystic Fibrosis		22
Gastrointestinal Diseases		20
	Chron disease	10
	Ulcerative Colitis	7
	Pancolitis	2
	Hirschsprung disease	1
Onco-Hematological Diseases		9
	Acute Lymphoblastic Leukemia	3
	Histiocytosis	1
	Hypothalamic Hamartoma	1
	Osteosarcoma	2
	Desmoplastic Small Round Cell Tumor	1
	Parietal Cavernoma	1
Migraines		151
Diabetes		19
Asthma		4
Scoliosis		4
Arthritis		2
Kidney Disease		7
	Chronic Kidney Failure	2
	Renal Dysplasia	1
	Renal Agenesis	1
	Nephrophysis	1
	Hemolytic Uremic Syndrome	1
	Glomerulonephritis Membranoproliferatin	1
Other Rare Diseases		13
	Duchenne Syndrome	1
	GLUT1 Deficiency Syndrome	1
	Schizotypic Disorder	1
	Primitive Sclerosing Cholangitis	1
	Atopic Dermatitis	1
	Coeliac Disease	1
	Schimke Immuno-Osseous Dysplasia	1
	Lupus	1
	Primary Hyperoxaluria Type I	1
	Thrombocytopenia	1
	Dravet Syndrome	1
	Janz Syndrome	1
	Optical Neuromyelitis	1

AIDS: Acquired Immune Deficiency Syndrome.

**Table 3:** The rates of different severities of depressive and anxiety symptoms (*n* = 334).

Variables	Depressive Symptoms (PHQ-9)		Anxiety Symptoms (GAD-7)		Comorbid Depression and Anxiety Symptoms	
	n	%	n	%	n	%
None	106	31.74	123	36.83	-	-
Mild	121	36.23	112	33.53	-	-
Moderate	79	23.65	66	19.76	-	-
Severe	27	8.08	32	9.58	-	-
Total symptoms	227	67.96	210	62.87	180	53.89

**Table 4:** Description of disease distributions, with significance determined by Wilcoxon’s signed-rank one-sample test.

Diagnosis			Anxiety Symptoms (GAD-7)		Depressive Symptoms (PHQ-9)	
	n	%	M	SD	M	SD
Migraines	151	45.21	7.81 ***	4.90	8.57 ***	4.62
Diabetes	19	5.69	9.21 **	5.45	9.89 **	5.92
Gastrointestinal Diseases	20	5.99	7.45 *	4.19	6.55	5.29
Epilepsy	53	15.87	6.94	5.25	6.23	4.97
Cystic Fibrosis	22	6.59	4.73	3.53	5.68	3.56
AIDS	16	4.79	4.87	3.04	6.80	5.36
Heart Diseases	14	4.19	4.71	4.18	5.07	4.57
Onco-Hemat. Diseases	9	2.69	6.78	3.53	6.56	2.74
Kidney Disease	7	2.09	8.29	6.16	5.57	4.12
Asthma	4	1.20	5.00	3.65	8.25	4.03
Scoliosis	4	1.20	3.75	2.75	3.25	1.50
Arthritis	2	0.60	2.50	2.12	3.50	3.54
Other Rare Diseases	13	3.89	7.85	4.41	8.23	7.05

*: *p*-value < 0.05; **: *p*-value < 0.01; ***: *p*-value < 0.001. Statistically significant results are marked in bold.

**Table 5:** Description of clinical and demographical distributions, with significance determined by Wilcoxon’s signed-rank one-sample test.

Variables				Anxiety Symptoms (GAD-7)			Depressive Symptoms (PHQ-9)		
		n	%	M	SD	Max Tot Score	M	SD	Max Tot Score
Overall		334	100	7.132 ***	4.83		7.505 ***	4.94	
Sex	Male	133	39.82	5.64	4.03	18 *	5.70	4.12	23 *
	Female	201	60.18	8.11 ***	5.06	21 *	8.69 ***	5.08	26 *
Pharmacological therapy	Yes	186	55.69	7.05 ***	4.88	21 *	7.20 ***	5.04	26 *
	No	148	44.31	7.24 ***	4.78	19 *	7.92 ***	4.80	22 *
Other (non-pharmacological) therapies	Yes	84	25.15	7.60 ***	4.73 *	18 *	8.13 ***	5.06	22 *
	No	240	71.86	7.02 ***	4.89	21 *	7.31 ***	4.85	26 *
Continuity of care during COVID-19 first lockdown	Yes	35	10.48	7.51 **	4.72	17 *	8.03 **	5.28	24 *
	No	293	87.72	7.11 ***	4.86	21 *	7.43 ***	4.61	26 *
Years of illness	≤1	48	14.37	8.15 ***	4.80	17 *	8.78 ***	5.75	24 *
	1 < 5	134	40.12	6.85 ***	4.91	21 *	7.70 ***	4.89	26 *
	≥5	111	33.23	6.82 ***	4.64	18 *	6.50 *	4.64	19 *
Age at diagnosis	Preschool	65	19.46	6.14	4.41	18 *	6.22	4.35	19 *
	School	227	67.96	7.29 ***	4.89	21 *	7.77 ***	5.14	26 *

*: *p*-value < 0.05; **: *p*-value < 0.01; ***: *p*-value < 0.001. Statistically significant results are marked in bold.

**Table 6:** Differences in mood scores between clinical and demographical sub-groups.

	W	df	*p*
**Age at diagnosis (preschool** **vs. school)** ^†^			
Tot score GAD-7	5432.000		0.073
Tot score PHQ-9	5553.000		0.039 *
**Pharmacological therapy (Y/N)** ^†^			
Tot score GAD-7	11094.000		0.217
Tot score PHQ-9	11592.500		0.051
**Other (non-pharmacological)** **therapies (Y/N)** ^†^			
Tot score GAD-7	6387.500		0.183
Tot score PHQ-9	6307.000		0.142
**Continuity of care during** **COVID-19 first lockdown (Y/N)** ^‡^			
Tot score GAD-7	−0.553	39.746	0.584
Tot score PHQ-9	−0.657	38.774	0.515
**Sex (m/f)** ^†^			
Tot score GAD-7	13,017.500		<0.001 ***
Tot score PHQ-9	13,522.000		<0.001 ***

*: *p*-value < 0.05; ***: *p*-value < 0.001. Note. ^†^: Mann–Whitney U-test; ^‡^: Welch’s *t*-test. Statistically significant results are marked in bold.

## Data Availability

The data presented in this study are available on request from the corresponding author. The data are not publicly available due to privacy and ethical reasons.
